# Phylogenetic Analysis of Nucleus-Encoded Acetyl-CoA Carboxylases Targeted at the Cytosol and Plastid of Algae

**DOI:** 10.1371/journal.pone.0131099

**Published:** 2015-07-01

**Authors:** Roger Huerlimann, Kyall R. Zenger, Dean R. Jerry, Kirsten Heimann

**Affiliations:** 1 College of Marine and Environmental Sciences, James Cook University, Townsville, Queensland, Australia; 2 Centre for Sustainable Tropical Fisheries and Aquaculture, James Cook University, Townsville, Queensland, Australia; 3 Comparative Genomics Centre, James Cook University, Townsville, Queensland Australia; Institut de Génétique et Développement de Rennes, FRANCE

## Abstract

The understanding of algal phylogeny is being impeded by an unknown number of events of horizontal gene transfer (HGT), and primary and secondary/tertiary endosymbiosis. Through these events, previously heterotrophic eukaryotes developed photosynthesis and acquired new biochemical pathways. Acetyl-CoA carboxylase (ACCase) is a key enzyme in the fatty acid synthesis and elongation pathways in algae, where ACCase exists in two locations (cytosol and plastid) and in two forms (homomeric and heteromeric). All algae contain nucleus-encoded homomeric ACCase in the cytosol, independent of the origin of the plastid. Nucleus-encoded homomeric ACCase is also found in plastids of algae that arose from a secondary/tertiary endosymbiotic event. In contrast, plastids of algae that arose from a primary endosymbiotic event contain heteromeric ACCase, which consists of three nucleus-encoded and one plastid-encoded subunits. These properties of ACCase provide the potential to inform on the phylogenetic relationships of hosts and their plastids, allowing different hypothesis of endosymbiotic events to be tested. Alveolata (Dinoflagellata and Apicomplexa) and Chromista (Stramenopiles, Haptophyta and Cryptophyta) have traditionally been grouped together as Chromalveolata, forming the red lineage. However, recent genetic evidence groups the Stramenopiles, Alveolata and green plastid containing Rhizaria as SAR, excluding Haptophyta and Cryptophyta. Sequences coding for plastid and cytosol targeted homomeric ACCases were isolated from *Isochrysis* aff. *galbana* (TISO), *Chromera velia* and *Nannochloropsis oculata*, representing three taxonomic groups for which sequences were lacking. Phylogenetic analyses show that cytosolic ACCase strongly supports the SAR grouping. Conversely, plastidial ACCase groups the SAR with the Haptophyta, Cryptophyta and Prasinophyceae (Chlorophyta). These two ACCase based, phylogenetic relationships suggest that the plastidial homomeric ACCase was acquired by the Haptophyta, Cryptophyta and SAR, before the photosynthetic Rhizaria acquired their green plastid. Additionally, plastidial ACCase was derived by HGT from an ancestor or relative of the Prasinophyceae and not by duplication of cytosolic ACCase.

## Introduction

Entering into an endosymbiotic relationship, in the form of an internal mutualist, offers the evolutionary advantage of gaining access to new biochemical pathways, resulting in increased competitiveness [1]. These endosymbiotic relationships provide many advantages and have shaped the world we know to a high degree. For example, the endosymbiosis between cnidarians and dinoflagellates has allowed corals to use the photosynthetic capacity of the endosymbiont to obtain additional energy. Of even greater importance were the more permanent primary endosymbiotic events which led to the evolution of mitochondria and chloroplasts (plastids) as organelles through the endosymbiosis of an alpha-proteobacterium and a cyanobacterium, respectively, enabling eukaryotes to prosper in an oxygen enriched atmosphere and to assimilate inorganic carbon through oxygenic photosynthesis [1]. Further endosymbiotic events involved the secondary endosymbiosis of a primary plastid containing photosynthetic eukaryote by a heterotrophic eukaryotic organism, leading to a diversification of photosynthesizing organisms containing secondary plastids [2]. The successful complete incorporation of an endosymbiont as an organelle requires the transfer of genes from the endosymbiont genome to the genome of the host, called endosymbiotic gene transfer. Furthermore, additional genes can be acquired through horizontal gene transfer from another organism or through gene duplication within an organism. This acquisition of genes by different methods from a variety of sources complicates the analysis of the evolutionary history of organisms and their genes. Acetyl-CoA carboxylase (ACCase) is such a gene with a complicated history that has not been investigated previously.

ACCase is a key enzyme involved in the highly conserved fatty acid synthesis pathway. Two forms of ACCase exist, the multi-subunit, prokaryotic, heteromeric form and the multi-domain, eukaryotic, homomeric form. The heteromeric form of ACCase is found in the cytosol of all prokaryotes (Table 1). In non-photosynthetic eukaryotes, the fatty acid *de novo* synthesis occurs in the cytosol and is facilitated by the nucleus encoded, homomeric ACCase (cytosolic ACCase) (Table 1). The *de novo* synthesis of fatty acids in organisms containing a plastid occurs in the plastid instead of the cytosol [2]. The plastids of organisms that arose from the primary endosymbiotic event (green and red algae, and plants) contain heteromeric ACCase, derived from the bacterial ancestor of the plastid (Table 1). Interestingly, there are two exceptions to this, the green algal group Prasinophyceae and certain plants (mainly the true grasses Poaceae), which contain a nucleus encoded homomeric ACCase in their plastids (plastidial ACCase) (Table 1, [2]). In contrast to the heteromeric ACCase containing primary plastids, all investigated organisms with secondary or tertiary plastids of eukaryotic origin contain a nucleus encoded, homomeric ACCase in their plastid (Table 1, [2]). In the following manuscript, cytosolic ACCase refers to nucleus encoded, homomeric ACCase expressed in the cytosol, while plastidial ACCase refers to nucleus encoded, homomeric ACCase expressed in the plastid.

**Table 1:** Gene and enzyme location of heteromeric and homomeric ACCases in prokaryotes, eukaryotes and plastid-containing eukaryotes (based on [2]).

	Heteromeric ACCase		Homomeric ACCase	
	Gene location	Enzyme location	Gene location	Enzyme location
**Prokaryotes**	Nucleoid	Cytosol	-	-
**Non-plastid containing eukaryotes**	-	-	Nucleus	Cytosol
**Eukaryotes containing a plastid derived from primary endosymbiosis**	Nucleus & plastidial genome	Plastid	Nucleus	Cytosol
**Eukaryotes containing a plastid derived from secondary or tertiary endosymbiosis**	-	-	Nucleus	Cytosol & Plastid

The plastidial heteromeric form of ACCase is clearly derived from the original cyanobacterial endosymbiont. Conversely, the origin of the plastid targeted homomeric form of ACCase in algae with secondary plastids is currently unknown. The three possible hypotheses on how the plastidial homomeric ACCase was acquired are: 1) endosymbiotic gene transfer from the cytosolic ACCase of the endosymbiont, 2) duplicated of the cytosolic ACCase from the host, 3) horizontal gene transfer from another organism. The first step in assessing these hypotheses is to place them into context with algal phylogeny.

Eukaryotic life is currently divided into six major supergroups, including Opisthokonta, Amoebozoa, Archaeplastida, Rhizaria, Chromalveolata and Excavata [3,4], of which the last four contain photosynthetic members. Endosymbiotic events were a driving factor in the evolution and diversification of photosynthetic organisms, especially algae [5]. From a parsimonious point of view, endosymbiotic events that successfully give rise to organelles are considered to be rare due to their complexity [6]. However, the observed algal diversity is difficult to explain in the most parsimonious way, and plastid diversity points to at least five endosymbiotic events, not including possible multiple endosymbiotic events in Dinoflagellata.

The Archaeplastida include the Viridiplantae (land plants and green algae), the Rhodophyta (red algae) and the Glaucocystophyta (a small group of freshwater microalgae) [7], all containing plastids surrounded by two envelope membranes. There is strong evidence that the plastids of these three groups evolved from a single primary endosymbiotic event involving a cyanobacterium [6]. Even though the primary endosymbiosis of a cyanobacterium is considered to only have occurred once, there is an euglyphid testate amoeba which has recently (in an evolutionary sense) taken up a cyanobacterium in what seems to be an independent primary endosymbiotic event (reviewed in [8]). In secondary endosymbiosis, a heterotrophic eukaryote took up a photosynthetic eukaryote containing a plastid derived from primary endosymbiosis. At least three secondary endosymbiotic events gave rise to a large number of highly diverse organisms with plastids derived either from a red or green alga. The supergroup Chromalveolata, first proposed by Cavalier-Smith [9], is composed of the red lineage joining the former kingdom Chromista (containing the Cryptophyta, Haptophyta and Stramenopiles) and infrakingdom Alveolata (comprising of Apicomplexa, Chromerida, Ciliophora and Dinoflagellata). In contrast, Chlorarachniophyta (Rhizaria) and Euglophyta (Excavata) have taken up their plastids in two independent endosymbiotic events from an ancestral core Chlorophyta (Ulvophyceae-Trebuxiophyceae-Chlorophyceae) and a Prasinophyceae, respectively [10,11]. This is supported by the former host organisms of Rhizaria and Excavata not being closely related, while there is a close phylogenetic relationship between all Chromalveolata [8,12]. If the plastidial homomeric ACCase shows a close relationship with the cytosolic homomeric ACCase of the host organism, it was likely derived from a gene duplication event. Conversely, a close relationship to the cytosolic ACCase of the endosymbiont suggests endosymbiotic gene transfer. Lastly, if there is no relationship to either, the gene was most likely derived through horizontal gene transfer.

Recently, the support for the Chromalveolata grouping has been waning [6]. While there is strong evidence that all Chromalveolates contain a red plastid from a secondary endosymbiotic event with a red alga, it is still controversial how many endosymbiotic events occurred [8,13]. Newer phylogenetic analyses group the Stramenopiles and Alveolata together with the Rhizaria (abbreviated as SAR), with Haptophyta as a sister group and the Cryptophyta being more closely related to the Viridiplantae [14]. This provides additional evidence against the monophyly of the Chromalveolata. In this case, a close relationship of the cytosolic homomeric ACCase sequences between the SAR species would support this grouping. Furthermore, a close relationship of the plastidial homomeric ACCase in the SAR species would support the acquisition and loss of a red plastid before the divergence of the Rhizaria. To further complicate matters, genes that are phylogenetically related to green algae have been found in diatoms (Stramenopile) [15], *Chromera velia* (Chromerida) [16] and numerous species of Stramenopile, Haptophyta and Cryptophyta [17,18]. However, a hypothesis that the red lineage contained an ancestral cryptic plastid of green origin has largely been refuted [12,19]. The presence of green algal genes in the Chromalveolata are mostly explained by either sample bias [19,20] or horizontal gene transfer [6] and their presence cannot be taken as proof for endosymbiotic gene transfer. Nevertheless, the single origin of Chromalveolata cannot explain all the findings of recent phylogenetic studies and less parsimonious solutions, including secondary and tertiary endosymbiosis, are invoked to explain the inconsistencies. For example, a single secondary endosymbiotic event was proposed, where an ancestral Cryptophyte took up a red alga, followed by a tertiary event that led to the Haptophytes, Stramenopiles and Alveolata [6]. This hypothesis also offers an explanation for the occurrence of heterotrophic members in the Chromalveolata, avoiding assumptions of multiple plastid losses [13].

In the present study, ACCase sequences were isolated from *Isochyris* aff. *galbana* (Haptophyta), *Chromera velia* (Chromerida) and *Nannochloropsis oculata* (Eustigmatophyceae), representing three taxonomic groups for which ACCase sequences were not yet well represented. These new sequences were incorporated with publicly available ACCase sequences to exhaustively examine the phylogeny of the plastidial and cytosolic form of homomeric ACCase and to integrate these findings with our current taxonomic understanding of algae. Phylogenetic analyses based on cytosolic ACCase were used to add information to the relationship of the hosts, while information of the plastidial ACCase aids to further unravel the origin of genes encoding enzymes targeted at plastids derived from secondary/tertiary endosymbiosis.

## Material and Methods

The sequencing and annotation of the acetyl-coA carboxylase genes for *Isochrysis* aff. *galbana*, *Chromera velia* and *Nannochloropsis oculata* used in this study has been described previously [21]. Sequence data for these sequences can be found in the GenBank data library under accession numbers: KF673096 to KF673101. The phylogenetic analysis was performed on amino acid sequences. Sequences not produced for this study were obtained from NCBI (www.ncbi.nlm.nih.gov) and JGI (genome.jgi-psf.org). Several partial ACCase sequences were excluded from the alignment (notably plastidial ACCase from *Emiliania huxleyi* and *Symbiodinium* Clade C and all sequences from *Ectocarpus siliculosus*). Accession numbers for the sequences used can be found in the supplemental material (S1 Table).

Sequences were aligned with MUSCLE (version 3.6; [22]) in Geneious Pro. GBlocks (version 0.91b; molevol.cmima.csic.es/castresana/Gblocks.html; [23,24]) was used to eliminate divergent regions and poorly aligned positions using standard settings. The resulting dimensions (number of taxa x number of amino acid positions) for the alignments were 55 x 564. The amino acid alignments are available on request. Models were tested and chosen with ProtTest (version 3.2; darwin.uvigo.es/software/prottest3; [25]) to find the best fitting model with AIC, using a maximum-likelihood (ML) starting tree. The most suitable model was LG+G+F. Bayesian inference (BI) and ML analyses were performed.

ML trees were performed in PhyML (version 3.0; www.atgc-montpellier.fr/phyml; [26]), using the models mentioned above with 4 rate categories, gamma estimated from the data and 2000 bootstraps.

Bayesian trees were performed in MrBayes (version 3.2; available from mrbayes.sourceforge.net; [27]) for 2*10^6^ generations, running 4 chains in parallel (3 heated) with a sampling frequency of 2,500 and a diagnostic frequency of 25,000. A four-category gamma model was used, as suggested by ProtTest, with the alpha parameter being estimated from the data during the run. The aa models were set to “mixed” to let Mr. Bayes determine the most suitable model, which was WAG+G. The average standard deviation of split frequencies was used to evaluate the convergence of the sampled chains and a 25% burn-in fraction was chosen for each analysis.

BI trees were used for the publication. FigTree (version 1.3.1; tree.bio.ed.ac.uk/software/figtree) was used to display finished trees, which were midpoint rooted.

## Results and Discussion

**Fig 1:**
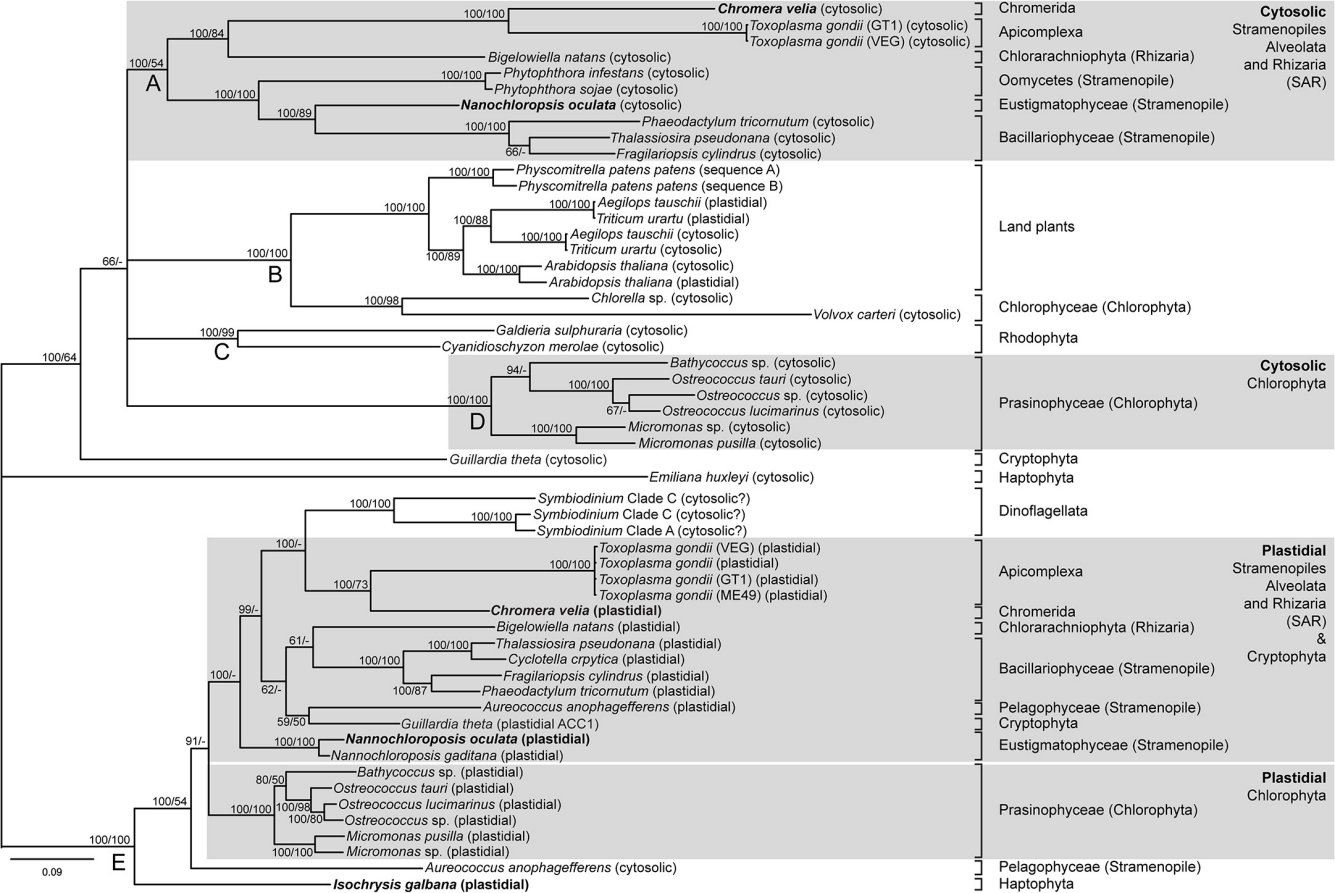
Phylogenetic consensus tree (MrBayes; WAG+G) based on 55 ACCase sequences and 564 amino acids positions. Cytosolic and plastidial ACCase are indicated. Sequences produced in this study are shown in bold. Statistical support for internal nodes was determined by Bayesian inference posterior probabilities (first, shown as % values) and bootstrap analysis for ML (second, Model LG+G+F). Only support values ≥50% are shown.

The Chromalveolate hypothesis is still hotly debated [6,28]. The traditional view fails to explain recent genetic evidence (e.g. the relationship of the SAR species to the exclusion of the Haptophyta and Cryptophyta), and the latest phylogenetic explanations invoke additional endosymbiotic events to explain inconsistencies [6]. In order to unravel the phylogenetic relationship of acetyl-CoA carboxylase (ACCase) between the different taxa, a phylogenetic consensus tree for plastidial and cytosolic ACCase was constructed using the Bayesian Inference (BI) and Maximum Likelihood (ML) methods (Fig 1). This dataset included all algal sequences of ACCase found on Genbank and JGI (accessed March 2013), to the exclusion of all the sequences of *Ectocarpus siliculosus*, the plastidial sequence of *Emiliania huxleyi* and the cytosolic sequence of one *Toxoplasma gondii* strain, which were incomplete and missed important binding regions. The sequence for the cytosolic ACCase of *Chromera velia* was also in three fragments, but included all four important binding regions (Table 2). Of the five major nodes (A to E), nodes B to E were strongly supported (BI = 100%, ML ≥ 99%), while node A was strongly supported by BI (BI = 100%, ML > 50%) (Fig 1). Node A, B, C and D contain cytosolic ACCase only, with the exception of the plastidial ACCase from plants. Conversely, node E contains mainly plastidial ACCase. Therefore, nodes A through D show the relationship of the hosts, while node E provides information on the origin of the plastidial ACCase.

Node A represents a major clade consisting of cytosolic ACCase of the Chromerida, Apicomplexa and Stramenopiles and a Chlorarachniophyte (Rhizaria) (Fig 1). The other two Chromalveolate taxa, *Guillardia theta* (Cryptophyta) and *Emiliana huxleyi* (Haptophyta), form an exception and are located outside of the five nodes. Within the SAR cluster, the relationship between the Chromerid *Chromera velia* and the Apicomplexan *Toxoplasma gondii* is well supported, and the more distant relationship with the Rhizarian *Bigelowiella natans* is identified, while the Stramenopiles form their own sub-clade. Based on these results, the proposed inclusion of the Stramenopiles, Alveolata and Rhizaria as SAR is supported, while the Cryptophyte and the Haptophyte are more distantly related [29,30]. However, ACCase-based phylogenies do not recover the close relationship of the Cryptophyte with the Archaeaplastida.

Node E contains all plastidial sequences of ACCase, with the exception of the land plants. Here *G*. *theta* (Cryptophyta) is nested strongly within the SAR species. In contrast to the phylogeny of plastidial GAPDH [31], plastidial ACCase of the haptophyte *Isochrysis galbana* is more basal to the SAR species, which agrees with a recent multi-gene study based on genomic DNA [14]. Surprisingly, the Prasinophyceae, which contain a plastid derived from a primary endosymbiotic event, are clustering strongly with the Chromalveolates, which contain a plastid derived from either a secondary or tertiary endosymbiotic event (Fig 1). This relationship was also identified using multi-gene analyses in Stramenopiles, Cryptophyta, and Haptophyta [15,17], where a strong association of certain genes with the Prasinophyceae has been found. This is in contrast to the true grasses, where an ancestral gene duplication event resulted in the functional expression of a homomeric, plastidial ACCase, coupled with a loss of heteromeric ACCase [32]. A separate gene duplication event, similar to the true grasses, occurred in the ancestor of *Arabidopsis thaliana*, resulting in a plastid-targeted copy of homomeric ACCase, which is expressed alongside the heteromeric ACCase, although at very low levels [32].

The close relationship between plastidial ACCases from SAR, Cryptophyta and Prasinophyceae shown here can either be explained by horizontal or endosymbiotic gene transfer, while gene duplication can be excluded as an explanation (Fig 1). This is in contrast to the replacement of the bacterial GAPDH gene found in the plastids of Archaeplastida with a eukaryotic GAPDH gene during the secondary endosymbiotic event that lead to the Apicomplexa, Rhodophyta, Dinoflagellata, Stramenopiles, and Haptophyta [33]. The replacement occurred through duplication of the cytosolic GAPDH, as is evident from the close phylogenetic relationship between the cytosolic and plastidial GAPDH in these taxa [33]. The presence of a cryptic green endosymbiont in the red lineage has been refuted [12,19], which makes an endosymbiotic gene transfer of ACCase unlikely. The strong relationship of the plastidial ACCase between the SAR species and the Prasinophyceae can therefore be explained by horizontal gene transfer from an unidentified organism, which could either be a Prasinophyte or an unsequenced alga related to the Prasinophyceae. The distant relationship of the cytosolic ACCase sequences of the SAR and Prasinophytes, in contrast, indicates that the ancestral hosts were only distantly related (Fig 1). The phylogenetic analysis of ACCase provides additional support for a serial secondary endosymbiotic event that gave rise to the green plastid containing Chlorarachniophyta (Rhizaria) within the SAR, This requires the loss of the red algal plastid and regain of a green plastid in the Chlorarachniophyta (see [6,12]). Even though this is less parsimonious, having acquired a plastid once could make subsequent acquisitions of plastids easier, similar to the case of the dinoflagellates [34].

A further point of interest in Node E is the clustering of the plastidial ACCases of *C*. *velia* and *T*. *gondii* and the ACCases of *Symbiodinium* (Fig 1). This supports the close relationship of *C*. *velia* with the Dinoflagellata and Apicomplexa [35]. Based on the α-CT binding motif, the *Symbiodinium* sequences were identified as cytosolic, however, they cluster strongly with the plastidial sequences of the SAR. Dinoflagellates are known to have complicated genomes, which could make it difficult to determine the localisation of the ACCase. Given the close relationship of *C*. *velia* and apicomplexan parasites, plastidial ACCase could be a potential target for drug development. *Chromera velia* could therefore be used as a substitute to screen compounds for the treatment of apicomplexan parasites, since it is easier to cultivate as is not dependent on a host [35]. ACCase inhibitors, often based on commercial herbicides acting on plastidial ACCase in the true grasses, have been investigated as potential drugs to treat apicomplexan infections and have shown promise in the reduction of the parasite load [36,37]. However, not all inhibitors showed the same activity [38]. The latter could be due to differences in the presence, localization and expression of ACCase between different species of apicomplexan parasites making a "one-size-fits-all" solution unlikely [39]. Furthermore, apicomplexan parasites are only dependent on *de novo* synthesis of FAs during their liver life stage (schizonts), while trophozoites (blood life cycle stage) are able to access plasma TAGs to supplement their FA needs [39,40], therefore limiting FA synthesis-based treatments to the liver life stage.

Node B is well supported and consists of the cytosolic and plastidial ACCase sequences of land plants and the cytosolic sequences of green algae (Fig 1). Within the land plants, the two sequences of the moss *Physcomitrella patens patens* form a close relationship. Furthermore, the plastidial and cytosolic ACCases of *Arabidopsis thaliana* form their own sub-clade, while the plastidial and cytosolic ACCaces of the true grasses (*Triticum urartu* and *Aegilops tauschii*) are clearly separated from each other, as well as from the sequences of *A*. *thaliana*. This demonstrates that the plastidial and cytosolic ACCases in land plants are paralogous within the true grasses and also within *A*. *thaliana*. Finally, the well supported node C consists of the cytosolic sequences of the two red algal species, while node D shows a single clade consisting of the cytosolic ACCase of the more ancient marine green Prasinophyceae (Fig 1).

## Conclusions

ACCase-based phylogenies can be used to investigate phylogenetic relationships in the highly diverse algae. Expanding on a previous study on the presence of homomeric ACCase in plastids derived from secondary/tertiary endosymbiosis [2], it is demonstrated here that the plastidial homomeric ACCase in the Chromalveolata and Rhizaria was derived from an ancestor or unsequenced relative of the green Prasinophyceae. Why the homomeric ACCase is preferred over the heteromeric ACCase in algae containing a plastid derived from secondary endosymbiosis is unclear. The case of *P*. *chromatophora* shows that in a primary endosymbiotic event, the heteromeric ACCase is preferred. However, during secondary symbiosis, targeting a single protein (i.e. homomeric ACCase) rather than several subunit peptides (i.e. heteromeric ACCase) to the plastid may be preferred [41]. Alternatively, there could be a general preference of replacing genes encoding plastid targeted enzymes present in the ancestor of the endosymbiont during secondary endosymbiosis, if other alternatives to the genes are available. This can be seen in the cases of ACCase (present study), glyceraldehyde-3-phosphate dehydrogenase [33] and fructose-1,6-bisphosphate aldolase [42] in the investigated SAR species, Haptophyta and Cryptophyta. In the case of ACCase, the plastidial ACCase sequence phylogenies cluster the Prasinophyceae with the SAR group, Haptophyta and Cryptophyta to the exclusion of the cytosolic ACCase, suggesting horizontal gene transfer. Why a new gene was acquired through HGT gene transfer, rather than gene duplication within the host remains to be elucidated.

The distinct lack of genomic data for many Phyla impedes the determination of the origin of genes that have been acquired through horizontal gene transfer. For example, the paraphyletic class Prasinophyceae, which are consistently associated with Chromalveolates, are currently only represented by the order Mamiellales (*Micromonas* sp., *Ostreococcus* sp. and *Bathyoccus* sp.). Information from the intervening green lineages may reveal that any seemingly unique ancestral acquisition of genes in the Chromalveolata are actually independently derived from multiple HGT events in different Chromalveolate lineages from intervening green lineages which have not yet been sequenced [43]. As more complete genome sequences become available, the complex picture of algal endosymbiosis will become clearer and the uncertainty about horizontal versus endosymbiotic gene transfer will be resolved for more genes.

## Supporting Information

S1 Table Homomeric ACCase sequence details. (PDF)
