# Supplementary material for: Phylogenetic Analysis of Nucleus-Encoded Acetyl-CoA Carboxylases Targeted at the Cytosol and Plastid of Algae
Source: PLoS One. 2015 Jul 1;10(7):e0131099. doi: 10.1371/journal.pone.0131099 (PMC4489017; doi:10.1371/journal.pone.0131099)
Supplement: S1 Table — (PDF) [file pone.0131099.s001.pdf]

S1 Table. Homomeric ACCase sequence details. Species names, taxonomy, protein identification, size, identification numbers and sources of sequences used for the phylogenetic analysis of homomeric ACCase.

Species	Taxonomy	Protein	Size	Identification	Source
Toxoplasma gondii	Apicomplexa	ACC1	2564	AAF04493	NCBI
Toxoplasma gondii	Apicomplexa	ACC2	1102	AAF04494	NCBI
Toxoplasma gondii (GT1)	Apicomplexa	ACCase	2252	EEE20751	NCBI
Toxoplasma gondii (ME49)	Apicomplexa	ACC1	2599	XP_002369961	NCBI
Toxoplasma gondii (ME49)	Apicomplexa	ACC2	3399	XP_002370640	NCBI
Toxoplasma gondii (VEG)	Apicomplexa	ACC1	2612	EEE30125	NCBI
Toxoplasma gondii (VEG)	Apicomplexa	ACC2	3373	EEE28725	NCBI
Cyclotella cryptica	Bacillariophyceae	ACCase	2089	AAA81471	NCBI
Fragilariopsis cylindrus (CCMP1102)	Bacillariophyceae	ACCase	2045	274583	JGI
Fragilariopsis cylindrus (CCMP1102)	Bacillariophyceae	ACCase	2302	145710	JGI
Phaeodactylum tricornutum	Bacillariophyceae	ACCase	2093	54926	JGI
Phaeodactylum tricornutum	Bacillariophyceae	ACCase	2283	55209	JGI
Thalassiosira pseudonana	Bacillariophyceae	ACCase	2089	6770	JGI
Thalassiosira pseudonana	Bacillariophyceae	ACCase	2251	12234	JGI
Arabidopsis thaliana	Brassicaceae	ACC1	2254	NP_174849	NCBI
Arabidopsis thaliana	Brassicaceae	ACC2	2356	NP_174850	NCBI
Physcomitrella patens patens	Bryophyte	ACCase	2238	EDQ80874	NCBI
Physcomitrella patens patens	Bryophyte	ACCase	2249	EDQ62113	NCBI
Bigelowiella natans	Chlorarachniophyta	ACC1	2031	92488	JGI
Bigelowiella natans	Chlorarachniophyta	ACC2	2053	18464	JGI
Bathycoccus prasinos	Chlorophyta	ACCase	2120	CCO17834	NCBI
Bathycoccus prasinos	Chlorophyta	ACCase	2177	CCO18633	NCBI
Chlorella sp. (NC64A)	Chlorophyta	ACCase	1868	56737	JGI
Micromonas pusilla (CCMP1545)	Chlorophyta	ACCase	2146	31755	JGI
Micromonas pusilla (CCMP1545)	Chlorophyta	ACCase	2094	16401	JGI
Micromonas sp. (RCC2999)	Chlorophyta	ACCase	2140	113382	JGI
Micromonas sp. (RCC2999)	Chlorophyta	ACCase	2190	104872	JGI
Ostreococcus lucimarinus	Chlorophyta	ACCase	2013	44400	JGI
Ostreococcus lucimarinus	Chlorophyta	ACCase	1994	11	JGI
Ostreococcus sp. (RCC809)	Chlorophyta	ACCase	2014	61393	JGI
Ostreococcus sp. (RCC809)	Chlorophyta	ACCase	2060	36970	JGI
Ostreococcus tauri	Chlorophyta	ACCase	2123	CAL50235	NCBI
Ostreococcus tauri	Chlorophyta	ACCase	1983	CAL56314	NCBI
Volvox carteri f. *nagariensis*	Chlorophyta	ACCase	2462	106840	JGI
Chromera velia	Chromerida	ACC1	2082	KF673098	Present study
Chromera velia	Chromerida	ACC2	-	KF673095-7	Present study
Guillardia theta	Cryptophyta	ACC1	2055	68771	JGI
Guillardia theta	Cryptophyta	ACC2	2171	72035	JGI
Nannochloroposis gaditana	Eustigmatophycea	ACC1	2139	AFJ69228	NCBI
Nannochloroposis oculata	Eustigmatophycea	ACC1	2,317	KF673100	Present study
Nannochloroposis oculata	Eustigmatophycea	ACC2	2,362	KF673101	Present study
Emiliana huxleyi (1516)	Haptophyta	ACCase	1577	455280	JGI
Emiliana huxleyi (1516)	Haptophyta	ACCase	2013	449545	JGI
Isochrysis aff. *galbana* (TISO)	Haptophyta	ACC1	2134	KF673099	Present study
Phytophthora infestans (T30-4)	Heterokontophyta	ACC	2326	EEY68805	NCBI
Phytophthora sojae	Heterokontophyta	ACC	2310	EGZ09435	NCBI
Aureococcus anophagefferens (1984)	Pelagophyceae	ACCase	1809	38836	JGI
Aureococcus anophagefferens (1984)	Pelagophyceae	ACCase	2153	65524	JGI
Ectocarpus siliculosus	Phaeophyta	ACCase	1848	Esi0198_0003	BOGAS
Ectocarpus siliculosus	Phaeophyta	ACCase	799	Esi0592_0001	BOGAS

Species	Taxonomy	Protein	Size	Identification	Source
Ectocarpus siliculosus	Phaeophyta	ACCase	1049	Esi0367_0023	BOGAS
Ectocarpus siliculosus	Phaeophyta	ACCase	166	Esi0403_0027	BOGAS
Aegilops tauschii	Poaceae	ACC2	2258	ACD46664	NCBI
Aegilops tauschii	Poaceae	ACC1	2311	ACD46679	NCBI
Triticum urartu	Poaceae	ACC2	2260	ACD46670	NCBI
Triticum urartu	Poaceae	ACC1	2311	ACD46677	NCBI
Cyanidioschyzon merolae	Rhodophyta	ACCase	2719	CMM188C	CMGP
Galdieria sulphuraria	Rhodophyta	ACC2	2482	EME32530	NCBI

Supplemental table 2 β-carboxyltransferase subunit of heteromeric ACCase Species names, taxonomy, protein identification, size, identification numbers and sources of sequences used for the phylogenetic analysis of the β-carboxyltransferase subunit of heteromeric ACCase.

Species	Taxonomy	Protein	Size	Identification	Source
Escherichia coli (K12)	Bacteria	β-CT	304	P0A9Q5	NCBI
Arabidopsis thaliana	Brassicaceae	β-CT	488	P56765	NCBI
Auxenochlorella protothecoides	Chlorophyta	β-CT	388	AEU08409	NCBI
Bryopsis hypnoides	Chlorophyta	β-CT	353	ACX33787	NCBI
Chara vulgaris	Chlorophyta	β-CT	293	ABA61965	NCBI
Chlorococcum humicola	Chlorophyta	β-CT	337	ADZ15145.1	NCBI
Chlamydomonas reinhardtii	Chlorophyta	β-CT	369	133238	JGI
Chlorella variabilis	Chlorophyta	β-CT	388	ADZ04992	NCBI
Chlorella vulgaris	Chlorophyta	β-CT	411	BAA57908	NCBI
Coccomyxa subellipsoidea (C-169)	Chlorophyta	β-CT	628	ADV29875	NCBI
Dunaliella salina	Chlorophyta	β-CT	349	ABO33321	NCBI
Helicosporidium sp.	Chlorophyta	β-CT	471	ABD33968	NCBI
Leptosira terrestris	Chlorophyta	β-CT	599	ABO69279	NCBI
Nephroselmis olivacea	Chlorophyta	β-CT	627	AF137379_28	NCBI
Oltmannsiellopsis viridis	Chlorophyta	β-CT	342	ABB81983	NCBI
Oocystis solitaria	Chlorophyta	β-CT	411	ACQ90742	NCBI
Parachlorella kessleri	Chlorophyta	β-CT	441	ACQ90907	NCBI
Pedinomonas minor	Chlorophyta	β-CT	381	ACQ90825	NCBI
Pseudendoclonium akinetum	Chlorophyta	β-CT	312	AAV80676	NCBI
Trebouxia aggregata	Chlorophyta	β-CT	515	ABX82657	NCBI
Trebouxiophyceae sp.	Chlorophyta	β-CT	622	AFQ93852	NCBI
Volvox carteri f. *nagariensis*	Chlorophyta	β-CT	478	82311	JGI
Anabaena variabilis (ATCC 29413)	Cyanobacteria	β-CT	316	YP_320706	NCBI
Microcystis aeruginosa NIES-843	Cyanobacteria	β-CT	316	BAG05575	NCBI
Nostoc azollae (0708)	Cyanobacteria	β-CT	317	ZP_03766652	NCBI
Huperzia lucidula	Lycopodiophyta	β-CT	316	AAT80716	NCBI
Paulinella chromatophora	Rhizaria	β-CT	288	ACB42560	NCBI
Cyanidium caldarium	Rhodophyta	β-CT	267	Q9TLW3	NCBI
Cyanidioschyzon merolae	Rhodophyta	β-CT	273	CMV207C	CMGP
Galdieria sulphuraria	Rhodophyta	β-CT	549	EME28527	NCBI
Gracilaria tenuistipitata var. liui	Rhodophyta	β-CT	293	AAT79592	NCBI
Porphyra purpurea	Rhodophyta	β-CT	288	P51198	NCBI
Porphyra umbilicalis	Rhodophyta	β-CT	288	AFC39868	NCBI
Porphyra yezoensis	Rhodophyta	β-CT	288	Q1XDT9	NCBI
Chaetosphaeridium globosum	Streptophyta	β-CT	297	AAM96498	NCBI
Chlorokybus atmophyticus	Streptophyta	β-CT	296	ABM87949	NCBI
Staurastrum punctulatum	Streptophyta	β-CT	307	AAX45681	NCBI
Zygnema circumcarinatum	Streptophyta	β-CT	297	AAX45794	NCBI
